# Dental Cement Placement to Temporize Recalcitrant Dental Pain From Acute Pulpitis

**DOI:** 10.1016/j.acepjo.2026.100349

**Published:** 2026-03-03

**Authors:** Brian Chinnock, Kevin Fortier

**Affiliations:** 1 Department of Emergency Medicine, University of California San Francisco ‐ Fresno Center for Medical Education and Research, Fresno, California, USA; 2 Private Practice, Anaheim, California, USA

**Keywords:** pulpitis, dental pain, dental caries, zinc oxide eugenol, dental cement

## Abstract

Acute pulpitis due to dental caries can commonly lead to severe pain that is recalcitrant to oral medications. Although definitive treatment normally requires a dentist, this is rarely available in the emergency department (ED), and patients can have prolonged delays for an available appointment. This case report described a patient with severe recalcitrant dental pain from acute pulpitis due to dental caries who had a temporary dental cement covering placed by the ED physician. The patient had almost complete resolution of dental pain that persisted for several days until the appointment with the dentist.

## 1 Introduction

Approximately 2% of emergency department (ED) visits in the United States are due to a dental complaint.^1^ Severe dental pain, described as a visual analog scale score of ≥7, has been described in 3 quarters of these patients presenting to the ED.^1^ This pain may be due to dental infection, such as a periapical abscess, but is commonly due to pulpitis. Pulpitis is an inflammation of the pulp cavity, usually due to exposure of the pulp to the oral cavity through carious decay, with subsequent pain, temperature, and pressure sensitivity due to involvement of nerves in the pulp.^2^ Definitive treatment for pulpitis, such as direct pulp capping and pulpectomy, requires a dentist, who is rarely available in the ED. The ED physician’s primary goal with these patients is to control pain until the patient can be seen by a dentist. Nonsteroidal anti-inflammatory drugs and acetaminophen may be helpful, but many patients who present to the ED have already been trying these medications.^3^ Although antibiotics are often prescribed, randomized, controlled trials have demonstrated they do not provide pain relief in patients without overt signs of infection (ie, facial swelling, intraoral abscess).^4^^,^^5^ Opioids are generally avoided due to lack of efficacy and potential for addiction. We presented the case of a patient with recalcitrant dental pain due to pulpitis from caries who had excellent prolonged pain relief with a temporary dental cement placed by the ED physician.

## 2 Case Report

**Figure 1:**
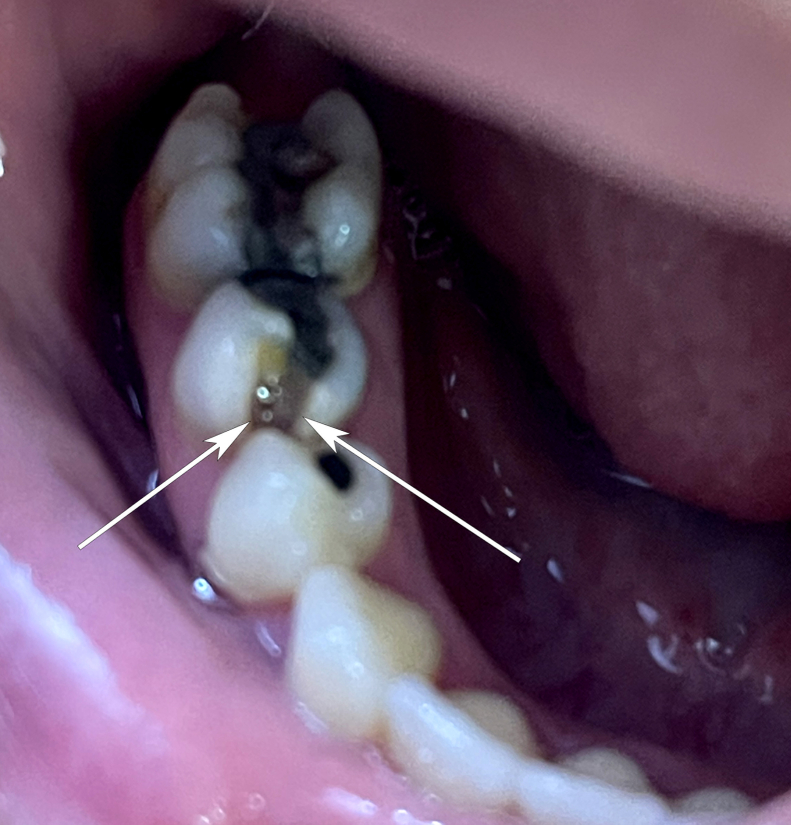
A carious defect of a molar with exposed pulp. The 2 arrows show the width of the carious defect and subsequent pulp exposure that extends vertically from the tooth’s occlusal surface to the gingiva.

A 33-year-old woman presented with a complaint of dental pain in the right lower first molar (tooth no. 30), which became severe and constant over the 4 days before the ED visit. Pain was described as 10 out of 10 but particularly worse with exposure of the tooth to hot or cold food and drink and with any pressure to the tooth. The patient was taking acetaminophen and ibuprofen at home with minimal relief. The soonest dental appointment was 3 days away. The physical examination of the patient revealed carious changes of the tooth, with a large vertical defect on the anterolateral (mesiobuccal) corner of the tooth, which extended from the occlusal surface of the tooth all the way to the gingiva. Pulp was visible (Fig 1). There was no facial or gingival swelling.

**Figure 2:**
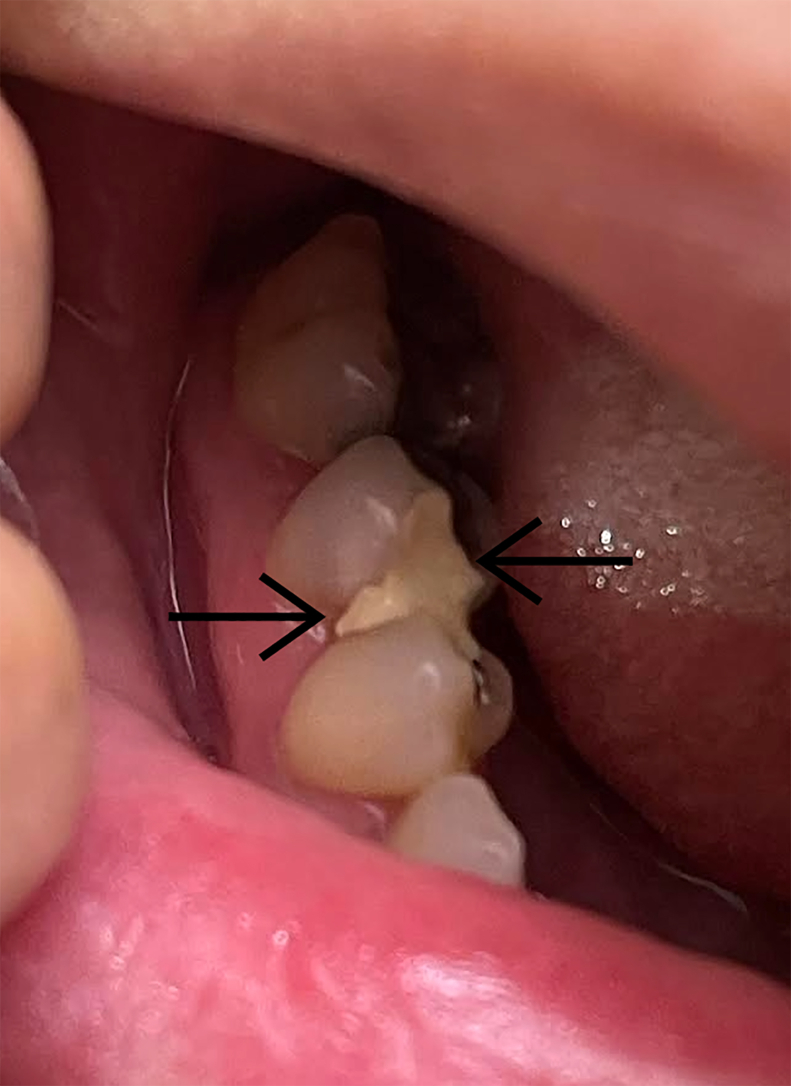
Dental cement covering of exposed pulp. The 2 arrows show the medial and lateral edges of the dental cement that has been applied, completely covering the exposed pulp of the carious defect.

With the examination findings, coupled with pressure/temperature sensitivity and lack of signs of overt infection, a provisional diagnosis of acute pulpitis due to dental caries was made. The patient received an inferior alveolar nerve block with 0.5% bupivacaine without epinephrine, which gave immediate pain relief. In addition, due to the inability to get immediate dental follow-up, we offered sealing of the exposed pulp with a temporary dental covering. The temporary cement used was zinc oxide/eugenol (ZOE; brand name Temrex CR Plus), which was available in the ED’s standard commercial emergency dental box (the Dental Box). For the dental covering procedure, the defect was first irrigated with a saline-flush syringe. The tooth was then blotted dry with gauze. The temporary cement was applied to the vertical defect with the applicator for about 30 seconds; the defect was completely covered, and then it was allowed to dry for 5 more minutes with the patient’s mouth open (Fig 2). About 10 minutes after the procedure, the patient described a return of the pain at 8 to 10 in severity. A block of the individual molar was done with 1 mL of bupivacaine using a 27-G needle inserted deep into the gingival sulcus, which gave complete pain relief.

The patient was discharged and subsequently had a dental follow-up 3 days later. At that appointment, the patient had another dental covering placed in preparation for a later root canal. On callback, the patient stated she had almost complete pain relief following the ED dental covering for the subsequent 3 days until she had her dental appointment. She also stated being able to sleep and eat almost normally, which she had not been able to do for the 4 days before that.

## 3 Discussion

This is the first published description of the use of a temporary dental cement by an ED physician to treat the pain resulting from acute pulpitis. Although the best initial treatment is definitive care by a dentist, at least one quarter of the US population may lack dental insurance coverage, with even higher rates of Medicare and Medicaid patients lacking coverage (approximately one-third).^6^^,^^7^ Even with coverage, the ability to get an acute care appointment may be limited.

In patients, such as in this case with dental pain without overt signs of infection, the most common cause is likely pulpitis. “Reversible pulpitis” is inflammation of the pulp, which should resolve after removal of the cause (most commonly the connection of the pulp cavity to the oral cavity caused by caries), whereas “irreversible pulpitis” is inflammation of the pulp that is incapable of healing and needs more invasive types of therapy like root canal therapy.^3^ A temporary covering of the tooth can seal off the pulp, prevent continued inflammation and pain of the pulp from direct contact with the oral cavity, and perhaps decrease the progression to irreversible pulpitis. Although oral pain medications can relieve pain to varying degrees, a case like this demonstrates how pain relief can be inadequate because the medications do not address the prevention of continued inflammation of the pulp.

The ZOE Temrex CR Plus was chosen in this case as it was one of the temporary coverings available in the Dental Box. This is a ZOE cement that comes in an automix-dual syringe that mixes together the 2 components as it is pushed through the syringe tip. It is described in the product information as able to be “used to perform tooth caps (fractures or cavities) or re-cement loose crowns” and has a setting time of only 90 seconds.^8^ Eugenol, the major constituent of oil of clove, has long been used in dentistry. It has profound anesthetic effects and both anti-inflammatory and antimicrobial activity.^9^ Despite these positive effects, there are concerns when it is placed directly in contact with exposed pulp, which is termed a “direct pulp cap.” When used as a direct pulp cap, as it was in this patient, there is more possibility of deleterious effects on the pulp by the dental covering material. ZOE, specifically the eugenol component, has been shown to have some pulp cytotoxicity. In addition, the ZOE starts to lose its pulp seal the longer it is in the mouth, although this may mean over many days and likely weeks.^10^ Finally, the only clinical study using ZOE as a direct pulp capping agent was done in 1949 and showed no pulp healing and no dentin formation when examined histologically after 12 weeks of placement; relief of pain was not measured.^11^

Although calcium hydroxide has been the gold standard in previous decades for pulp capping, it was not used in our case because it is not part of the standard kit and has to be ordered separately. Disadvantages of its use include the need to manually mix the catalyst and base before application.

Despite its disadvantages, ZOE is still a reasonable choice as a temporary pulp covering for the ED physician to use for a patient with acute pulpitis due to caries. First, ZOE’s pulp cytotoxicity is described in dental literature in comparison to other dental compounds, which are not cytotoxic. However, the proper comparison for ZOE in the ED is the patient who receives no pulp covering and has continued pulp exposure for many days while awaiting dental follow-up. This comparison has not been made, and the risk of continued pulp exposure certainly increases the possibility of necrosis and irreversible pulpitis. Second, the dental literature does not describe any obvious deleterious effects from ZOE, such as increased infection rate, pain, or inability to perform future procedures.^10^ Third, the pulp cytotoxicity described in the dental literature is only described in in-vitro and animal studies. The temporary increase in pain that occurred after the placement of the pulp covering would not be unusual, as any procedure near the pulp chamber can trigger short-term inflammation. Anti-inflammatory medication, if the pain is mild, or another block if the pain is more severe, as in this patient, can help decrease the pain while the inflammation calms down.

Although our patient seemed to benefit from the dental covering, it is possible that this pain relief occurred by chance. In addition, covering the defect still may not decrease inflammation of the pulp in some patients, and could even increase inflammation and pain. A randomized controlled trial showing superiority of pain relief with dental covering by the ED physician vs placebo would be indicated before recommending this as standard of care.

## 4 Summary

This case report described a common presentation of a patient with dental pain from pulpitis due to dental caries who is failing outpatient treatment with acetaminophen and ibuprofen. Use of a temporary cement, ZOE, enabled excellent pain relief, temporizing the patient until the time of the dental appointment. A future randomized, controlled trial would be helpful to determine the efficacy of dental coverage in this group of patients and potentially add an important mode of treatment for these ED patients, whose pain is commonly severe and difficult to control.

## Funding and Support

By *JACEP Open* policy, all authors are required to disclose any and all commercial, financial, and other relationships in any way related to the subject of this article as per ICMJE conflict of interest guidelines (see www.icmje.org). The authors have stated that no such relationships exist.

## Conflict of Interest

All authors declared that they have no known competing financial interests or personal relationships that could have appeared to influence the work reported in this paper.
